# Posttraumatic Compressive Neuropathy of the Deep Motor Branch of the Ulnar Nerve Caused by Heterotopic Ossification

**DOI:** 10.1016/j.jhsg.2024.02.001

**Published:** 2024-03-27

**Authors:** Kali N. Stevens, Kevin J. Malone

**Affiliations:** ∗ Department of Orthopaedic Surgery, University Hospitals Cleveland Medical Center, Cleveland, OH

**Keywords:** Claw hand, Compressive neuropathy, Deep motor branch of ulnar nerve, Heterotopic ossification, Intrinsic weakness

## Abstract

We report a case of posttraumatic compressive neuropathy of the deep motor branch of the ulnar nerve occurring in the setting of heterotopic ossification following a direct blow to the hypothenar eminence. Over several weeks, the patient developed ring and little finger claw deformities and atrophy of his first dorsal interosseous and adductor pollicis muscles with sparing of sensation. Electromyography and nerve conduction study localized the area of injury, and computed tomography confirmed the presence of heterotopic bone near the deep motor branch of the ulnar nerve. Intraoperatively, the deep motor branch of the ulnar nerve was under tension as it traversed volarly over the hook of hamate and heterotopic bone. Decompression of the deep motor branch of the ulnar nerve with resection of heterotopic bone and the hook of hamate was performed. Six months postoperatively, the patient demonstrated resolution of clawing and improving strength of his ulnar nerve innervated intrinsic muscles.

Compression of the ulnar nerve at the cubital tunnel is the second most common peripheral compressive neuropathy; however, compression at the distal ulnar tunnel of the wrist occurs relatively infrequently.^1^ The distal ulnar tunnel is classically divided into three zones in which compression can occur based on structures comprising the tunnel.^2^^,^^3^ Zone 1 is the portion of the tunnel extending from the proximal edge of the palmar carpal ligament to the bifurcation of the ulnar nerve. Zone 2 contains the deep motor branch of the ulnar nerve and spans from the bifurcation of the ulnar nerve to the fibrous arch of the hypothenar muscles. Zone 3 also begins just distal to the bifurcation of the ulnar nerve and contains the superficial branch of the ulnar nerve. Compression in these zones leads to combined motor and sensory deficits, intrinsic muscle weakness, and isolated sensory deficits, respectively.^2^^,^^3^ The pisohamate hiatus is a secondary compression site of the deep motor branch of the ulnar nerve, bound deeply by the pisohamate ligament and superficially by the fibrous arch of the hypothenar muscles connecting the pisiform to the hook of hamate.^3^^,^^4^ Causes of compression at the distal ulnar tunnel include acute or repetitive trauma, hook of hamate fracture or nonunion, anomalous muscles, or space-occupying lesions such as ganglions, thromboses, and pseudoaneurysms.^1^

Heterotopic ossification is a pathologic process in which extraskeletal bone is formed in muscle and soft tissues.^5^ It is abnormal tissue healing in which injury leads to an inflammatory reaction with inappropriate activation of osteogenesis or osteochondrogenesis at an extraosseous site.^5^ Heterotopic ossification is a known complication of trauma, surgery, and other local or systemic insults.^5^ Heterotopic ossification lesions can be small and inconsequential or cause significant morbidity.^5^ We present a case report of a patient with posttraumatic compressive neuropathy of the deep motor branch of the ulnar nerve caused by heterotopic ossification.

## Case Report

### Patient presentation and diagnostic studies

A 43-year-old left-handed man with a history of type 2 diabetes mellitus and hypertension presented for evaluation of abnormal posturing and control of his left ring and little fingers. He initially denied injury; however, on further questioning, he recalled being kicked in the hand by an aggressive student while working as a school principal. This direct blow to the base of his left palm on the hypothenar eminence occurred approximately 3–4 weeks prior to onset of symptoms. He denied any sensory disturbances.

He was seen by a hand surgeon who evaluated him for possible flexor pulley rupture versus central slip incompetence. Magnetic resonance imaging of the left hand revealed no evidence of pulley rupture or flexor or extensor tendon abnormalities. Over the ensuing weeks, he began to demonstrate claw deformities of the ring and little fingers and then developed atrophy of his first dorsal interosseous and adductor pollicis muscles with weakness of grip and pinch. An electromyography and nerve conduction study was performed, and the results were consistent with injury to the distal deep motor branch of the ulnar nerve with sparing of both the abductor digiti minimi muscle and sensory involvement. He was referred to occupational therapy and had some improvement in function with the use of a lumbrical bar splint; however, his symptoms remained unchanged. He subsequently saw another hand surgeon, and no additional treatment recommendations were provided.

**Figure 1:**
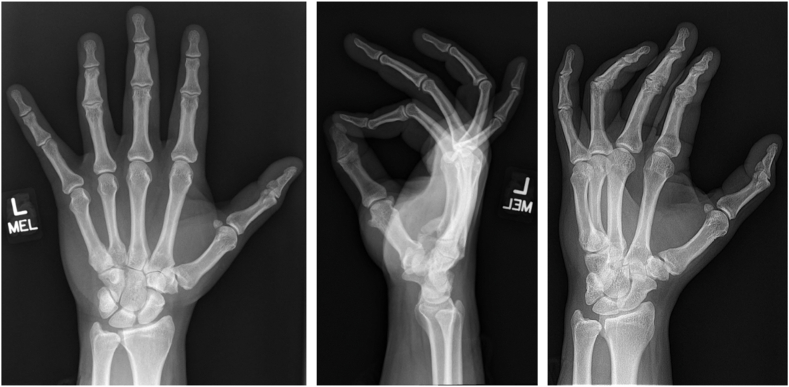
Posteroanterior, lateral, and oblique radiographs of the left hand obtained 2 months after injury demonstrating heterotopic bone arising from the volar base of the little finger metacarpal extending proximally toward the hook of hamate. There is also a small avulsion fracture at the volar base of the middle phalanx of the middle finger and evidence of a remote thumb metacarpophalangeal joint radial collateral ligament injury.

**Figure 2:**
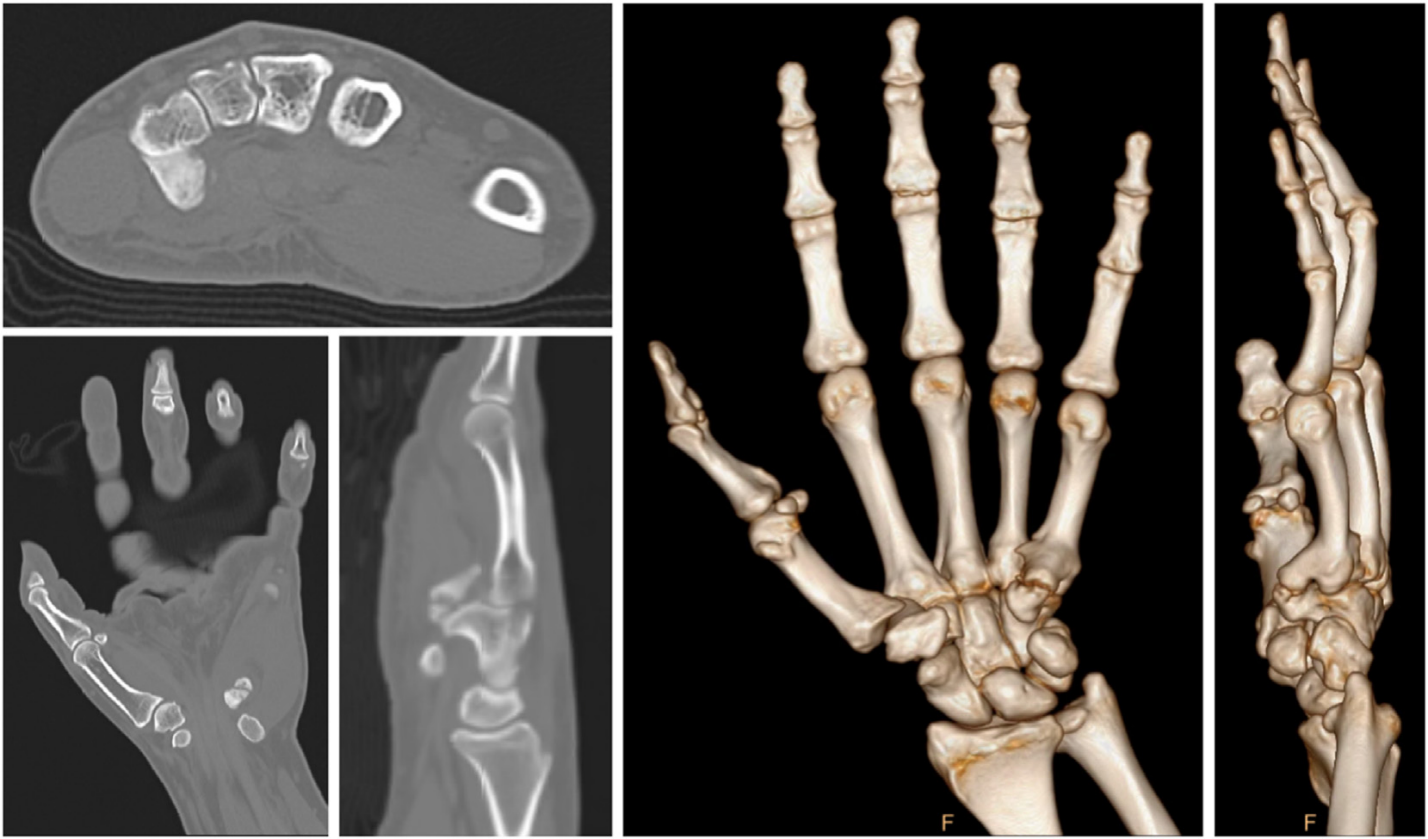
Computed tomography scan and three-dimensional reconstructions of the left hand obtained 6 months after injury demonstrating heterotopic bone formation between the hook of hamate and volar little finger metacarpal base.

The patient presented to our hand surgery clinic 6 months after injury. Previously obtained radiographs of the left hand were reviewed, which revealed heterotopic bone arising from the volar base of the little finger metacarpal extending proximally toward the hook of hamate (Fig. 1). Computed tomography scan of the left hand was then obtained, which confirmed the presence of abnormal bone formation near the deep motor branch of the ulnar nerve (Fig. 2). This raised concern for posttraumatic compressive neuropathy of the deep motor branch of the ulnar nerve caused by heterotopic ossification.

### Surgery

Two weeks after presentation, the patient was taken to the operating room for decompression of the deep motor branch of the ulnar nerve with resection of heterotopic bone and hook of hamate. A Bruner incision was made extending from the radial border of the flexor carpi ulnaris tendon in the distal forearm distally over the hypothenar eminence toward the fifth metacarpal. The ulnar artery was identified and traced into the palm, where the superficial palmar arch was mobilized in a radial direction. The ulnar nerve and its divisions were identified within the palm and dissected free from surrounding muscle. The hypothenar motor branches and branches to the fourth web space and the ulnar border of the little finger were preserved.

**Figure 3:**
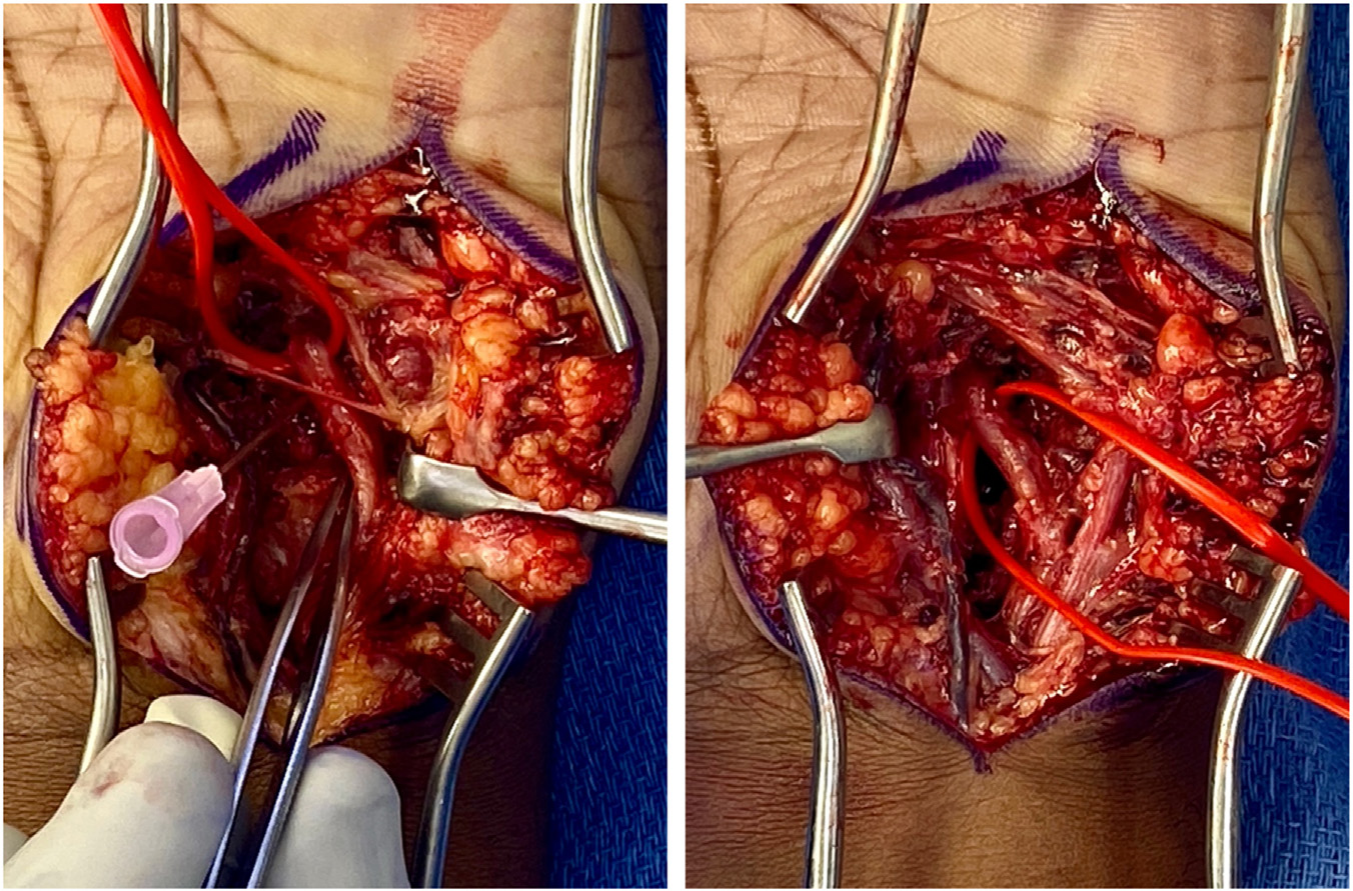
Intraoperative photographs. Left: the deep motor branch of the ulnar nerve (vessel loop) was translated volarly and was under tension as it passed superficial to the hook of hamate (needle) and heterotopic bone located just distal to the hook of hamate. Right: the deep motor branch of the ulnar nerve was decompressed following release of the transverse carpal ligament and resection of the prominent volar portions of heterotopic bone and the hook of hamate.

**Figure 4:**
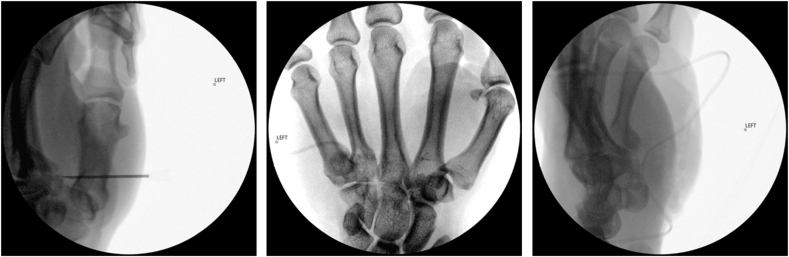
Intraoperative fluoroscopic images. Left: needle localizing the hook of hamate underlying the deep motor branch of the ulnar nerve. Center and right: anteroposterior and lateral views following excision of prominent heterotopic bone and the volar half of the hook of hamate.

The deep motor branch of the ulnar nerve appeared volarly translated, essentially located superficial to the hook of hamate (Fig. 3). Intraoperative fluoroscopy was used to confirm the position of the hook of hamate as well as heterotopic bone just distal to it (Fig. 4). The deep motor branch was traced distally and appeared hypertrophic and edematous. The transverse carpal ligament was released from the radial aspect of the hook of hamate and from the heterotopic bone. This allowed for radial retraction of the flexor tendon contents of the carpal tunnel and exposure of the deep motor branch of the ulnar nerve. It was draped over the heterotopic bone and dove deep underneath the flexor tendons. The ulnar nerve was completely decompressed and was mobilized to gain circumferential access to the heterotopic bone and hook of hamate. The prominence of the heterotopic bone as well as the volar 50% to 60% of the hook of hamate were carefully removed using rongeurs. Following resection of these structures, motion was visualized at the fifth carpometacarpal joint. Clinically, the ulnar nerve and its branches remained intact, and there was no further evidence of tension or compression on the deep motor branch of the ulnar nerve.

### Postoperative course

**Figure 5:**
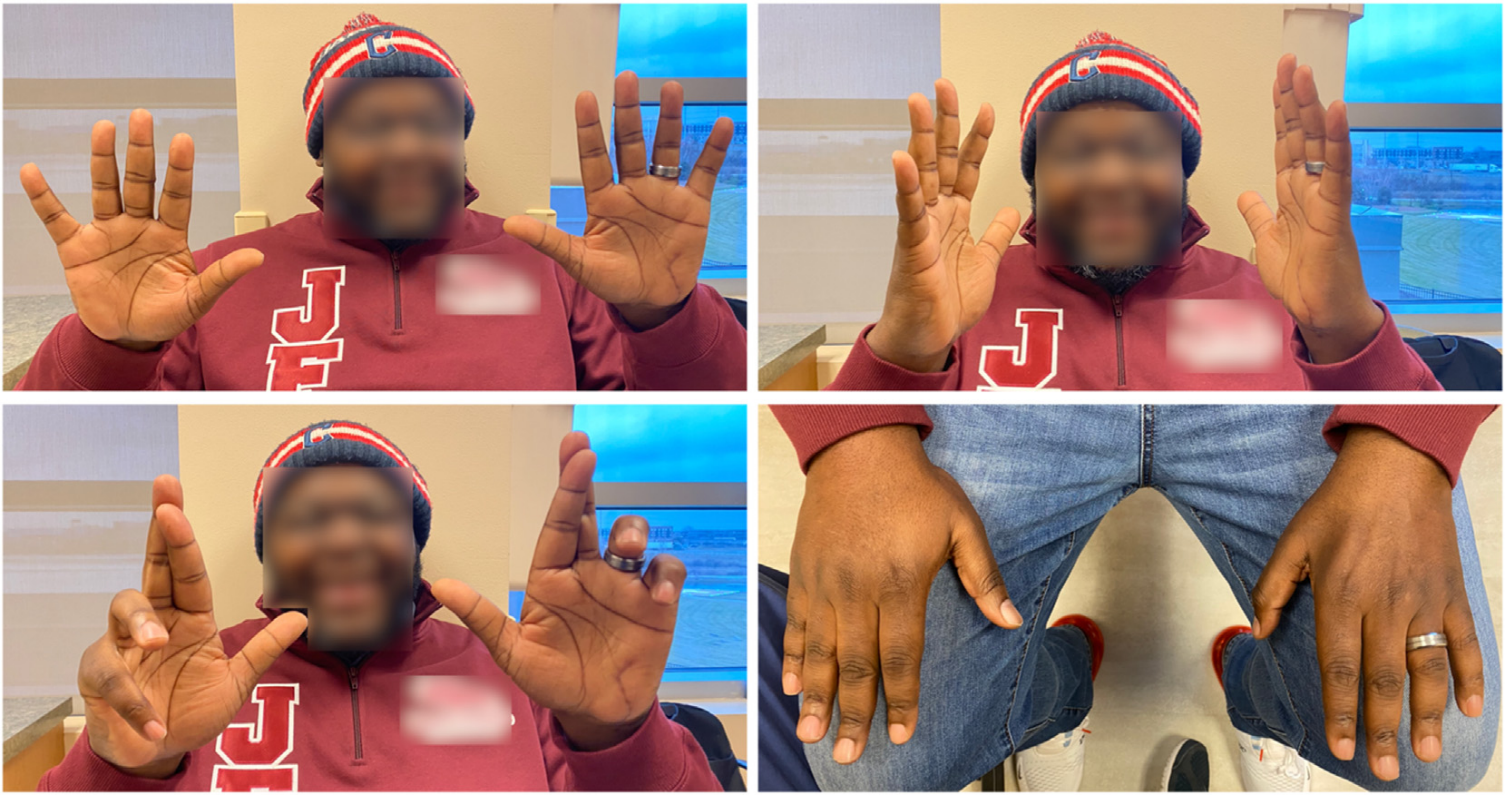
Clinical examination photographs taken 6 months postoperatively, demonstrating resolution of the patient’s clawing and ability to abduct and adduct digits of the left hand. There is persistent atrophy in the first web space and resolution of intermetacarpal atrophy.

The patient was seen for routine postoperative visits to monitor for signs of reinnervation of his ulnar nerve innervated intrinsic muscles. At 2 weeks, his surgical incision was healed, and examination findings were otherwise unchanged compared with preoperative findings. He was referred to occupational therapy for range of motion exercises, intrinsic muscle strengthening, and fabrication of an anticlaw orthosis. At 6 weeks, the patient reported subjective improvement in hand strength; however, he demonstrated persistent ring and little finger clawing. He continued formal occupational therapy for 3 months, at which time he transitioned to a home therapy program. Six months postoperatively, the patient returned for follow-up and was very pleased with his progress. On examination (Fig. 5), he had no clawing and was able to demonstrate full coordinated finger flexion and extension. He had reasonable finger abduction (better abduction of ulnar digits than radial digits), some persistent first web space atrophy, and resolution of preoperative intermetacarpal atrophy. Grip and pinch strength were slightly weaker on the left compared to the uninjured hand. The patient will be followed for further recovery of his first dorsal interosseous and adductor pollicis muscles; however, he currently wishes to avoid another operation with the anticipation that his pinch strength will continue to improve.

## Discussion

Heterotopic ossification has been described as a cause of peripheral neuropathy including compression of the ulnar nerve proper. Fikry et al^6^ published a case series of eight patients who sustained head injuries and developed elbow ankylosis and associated cubital tunnel syndrome due to ulnar nerve entrapment in heterotopic bone. Ulnar neuropathy has also been reported due to heterotopic ossification at the elbow after stroke and burns that caused encircling of the ulnar nerve within a bony tunnel.^7^^,^^8^ Posttraumatic myositis ossificans at the medial elbow has been identified as the cause of compressive neuropathy of the ulnar nerve in a multiply injured patient involved in a traffic accident.^9^ Finally, heterotopic ossification that formed in the distal forearm following revision carpal tunnel release has been described as causing mixed ulnar and median nerve compression symptoms.^10^

To our knowledge, this is the first reported case of heterotopic ossification causing compression of the deep motor branch of the ulnar nerve. Given the patient’s clinical signs of claw deformity of the ring and little fingers and atrophy of his first dorsal interosseous and adductor pollicis muscles with sparing of sensation, we had a high index of suspicion for a pathologic process within zone 2 of the distal ulnar tunnel of the wrist. Interestingly, the patient’s acute traumatic injury did not result in an acute nerve injury, but rather in a gradual onset of weakness in ulnar nerve innervated intrinsic muscles associated with increased pressure exerted by the formation of heterotopic bone. Decompressing the patient’s deep motor branch of the ulnar nerve allowed for spontaneous nerve recovery and reinnervation of his ulnar nerve innervated intrinsic muscles, predictably starting with restoration of function of his hypothenar and more ulnar interosseous muscles as well as his third and fourth lumbricals. Full restoration of the patient’s pinch strength remains to be determined as his nerve recovery proceeds.

## Informed Consent

Informed consent was obtained from the patient for publication of this case report and accompanying images according to the CAse REport (CARE) guidelines.

## Conflicts of Interest

No benefits in any form have been received or will be received related directly to this article.

## References

[1] Patterson J.M.M., Novak C.B., Mackinnon S.E., Wolfe S.W., Pederson W.C., Kozin S.H., Cohen M.S. (2022).

[2] Gross M.S., Gelberman R.H. (1985). The anatomy of the distal ulnar tunnel. Clin Orthop Relat Res.

[3] Maroukis B.L., Ogawa T., Rehim S.A., Chung K.C. (2015). Guyon canal: the evolution of clinical anatomy. J Hand Surg Am.

[4] Hayes J.R., Mulholland R.C., O’Connor B.T. (1969). Compression of the deep palmar branch of the ulnar nerve. Case report and anatomical study. J Bone Joint Surg Br.

[5] Meyers C., Lisiecki J., Miller S. (2019). Heterotopic ossification: a comprehensive review. JBMR Plus.

[6] Fikry T., Saidi H., Madhar M., Latifi M., Essadki B. (2004). Cubital tunnel syndrome and heterotopic ossification. Eight case reports. Article in French. Chir Main.

[7] Özişler Z., Yalçın E., Özel S. (2018). Ulnar neuropathy due to heterotopic ossification: a rare complication after stroke. Turk J Phys Med Rehabil.

[8] Wu Y., Liu M., Qu W. (2019). ‘Bony’ cubital tunnel syndrome caused by heterotopic ossification. Br J Neurosurg.

[9] Choi K.H., Park S.G., Baek J.H., Lee W., Chang M.C. (2021). Myositis ossificans causing ulnar neuropathy: a case report. J Int Med Res.

[10] Maheshwari A.V., Dua K., Wham B., Kahila M., Kolla S., Stracher M.A. (2022). Heterotopic ossification after revision carpal tunnel release causing mixed ulnar and median compression neuropathy. J Hand Surg Am.

